# Behavioral Barriers to Digital Health and Conditions of Digital Resilience Among Older Adults: Evidence from China

**DOI:** 10.3390/bs16091550

**Published:** 2026-09-01

**Authors:** Yu Ren, Weihua Yang, Wan Li

**Affiliations:** School of Management, Dalian Polytechnic University, Dalian 116034, China; 240711202000978@xy.dlpu.edu.cn

**Keywords:** digital health, older adults’ behavioral barriers to digital health, individual digital risks, policy-supported digital resilience, behavioral public policy, COM-B model, policy text analysis

## Abstract

As health services shift to digital delivery, older adults face practical online risks, including operational errors, difficulty interpreting clinical information, unclear privacy consent procedures, and telecommunications fraud. Existing empirical work mostly adopts questionnaire and interview designs to document older adults’ real-world digital usage, yet few studies systematically analyze policy documents as an external intervention system and assess how they address four core usage barriers: limited digital proficiency, constrained service access, weak sustained participation, and individualized online safety threats. This paper analyzes 1008 policy records extracted from the Peking University Legal Database covering 2007–2025, and develops a four-dimensional analytical framework split into B1 (Digital Capacity and Age-Friendly Support), B2 (Digital Health Management Support), B3 (Home- and Community-Based Care Support), and B4 (Institutional Care and Risk Governance). The analytical toolkit combines a Capability–Opportunity–Motivation–Behavior (COM-B) diagnostic model, an M1–M4 intervention-mechanism coding protocol, Latent Dirichlet Allocation (LDA) topic modeling, and two indices: the Digital Adaptation Policy Orientation Index (DAPOI) and the Behavioral Intervention Diversity Index (BIDI). Coding outputs reveal a pronounced structural imbalance in policy allocation for older adults’ digital health in China: community and institutional governance clauses (B3, B4) dominate the corpus, while person-centred support including one-on-one operational coaching, clinical data interpretation guidance and offline manual backup channels accounts for a marginal share of all coded entries (B1, B2). Age-friendly digital policies issued circa 2020 serve primarily short-term emergency adjustments instead of permanent institutional arrangements. Built on full-text policy coding outputs, this work defines policy-supported digital resilience as a holistic protective system formed by coordinated institutional clauses. Every result reported is sourced exclusively from supply-side policy records, which provides policy-level evidence for subsequent behavioral research on older adults’ digital risk management and digital welfare construction.

## 1. Introduction

Online appointment, telemedicine and chronic disease monitoring tools are now widely adopted across China’s medical system. However, merely building up digital facilities cannot deliver practical health improvements to older adults. Opening online service entry points only provides preliminary access; whether older adults can gain real medical advantages depends on four key capabilities: proficient digital operation, accurate comprehension of medical materials, trust in online medical systems, and safe participation in remote diagnosis. A full set of digital medical workflows covers online booking, electronic medical insurance certificates, telehealth visits and chronic disease tracking, alongside privacy authorization procedures. These daily digital operations bring four major types of challenges to older users: insufficient operational skills, limited accessible service channels, low willingness to keep using such tools, and constant risks of personal information and property leakage (World Health Organization, 2021). Such difficulties appear in every medical link, ranging from outpatient booking and bill payment to remote treatment and long-term illness monitoring, as well as older adult care service registration (World Health Organization, 2020). As observed in relevant studies, older adults living with chronic illnesses usually cannot make sense of monitoring data and abnormal physical reminders (Jiang et al., 2024); groups with less formal education struggle to understand complicated privacy agreements and online payment rules; older adults living alone will refuse digital medical services out of worry over telecom fraud (S. Kim et al., 2025). A large body of research on electronic health literacy confirms that most older adults lack competence in equipment operation, health information searching, identifying false medical content and consistent online service use (Xie & Mo, 2023; Dong et al., 2023). While digital skill courses can ease such troubles to a certain extent, such training only works when it offers repeated offline practice combined with authentic medical service cases (Sardareh et al., 2024; Pinheiro et al., 2026).

The severity of digital hardship varies markedly across older adult subgroups. Rural-dwelling older adults face compounded constraints from inadequate network infrastructure and geographical service remoteness; less-educated older adults encounter formidable obstacles in multi-step, terminology-laden online workflows (Li et al., 2024); those living alone lack accessible peer or family assistance; and disabled or semi-disabled individuals depend on surrogate agents or offline manual alternatives (Choi et al., 2022). Existing cohort studies confirm that educational attainment, household income, urban–rural residential location, physical functionality, family support networks, and platform age-friendly design collectively shape the magnitude of the digital divide among older adults (Chadwick et al., 2024). Consequently, merely launching digital health platforms without complementary supportive mechanisms tends to widen the gap between nominal service availability and practical usability for aging populations.

Prior research has extensively investigated individual digital barriers experienced by older adults through surveys and qualitative interviews. Less attention has been paid to how multi-year policy records recognize, prioritize, and respond to these risks at the institutional level (Money et al., 2024; Leong et al., 2024; Knotnerus et al., 2024). Research on aged-care policies tends to treat community care, institutional care, and medical–elderly service integration as separate domains, yielding fragmented insights that fail to connect digital operability, health management, and offline backup support (Lu & Wei, 2021; Jin et al., 2021). Moreover, most applications of the COM-B (Capability–Opportunity–Motivation–Behavior) model remain confined to individual-level interventions and experimental designs, rather than scaling up to quantitative policy-text coding. This study addresses these research gaps by constructing a unified four-dimensional analytical framework that bridges individual behavioral constraints and macro-level policy supply, thereby extending the applicability of behavioral models to institutional-text analysis (Michie et al., 2011).

Grounded in behavioral public policy theory, this study establishes an analytical linkage between older adults’ practical difficulties and top-down policy provisioning. Rather than applying the COM-B model solely to intervention or service-design experiments, we repurpose it as a diagnostic instrument for policy-document evaluation. Specifically, we first identify older adults’ constraints related to capability, opportunity, motivation, and digital risk exposure, and then assess whether corresponding external supports are embedded within existing policy instruments. The objective is not mere policy categorization, but a targeted examination of whether institutional provisions adequately address older adults’ authentic dilemmas in medical and chronic-disease management contexts (Koulouvari et al., 2025; Chater & Loewenstein, 2023).

Adopting older adults’ digital vulnerability as the analytical point of departure, this paper systematically reviews the multi-year external intervention system shaped by policy promulgations, and explores the health safeguard conditions constructed through institutional supply. Four complementary dimensions of policy support are disaggregated: digital capacity cultivation, service opportunity provision, willingness enhancement, and risk mitigation. Digital medical services deliver real value to older adults not merely by offering online service portals, but by lowering usage barriers, clearing confusing medical information and removing institutional access gaps. Solving these three pain points is critical to safeguarding older adults’ overall physical and mental health.

## 2. Literature Review and Theoretical Framework

### 2.1. A COM-B Framework Analysis of Digital Usage Barriers Faced by Older Adults

Adequate digital operation skills form the basic prerequisite that allows older adults to make use of online medical resources. Such skills cover far more than simple mobile phone operations, including app setup, real-name authentication, retrieval of electronic medical certificates, analysis of chronic disease monitoring data, identification of false health content, and comprehension of privacy clauses (Schirmer et al., 2023). Frequent software version upgrades repeatedly raise learning barriers for users. Older adults with advanced age or low educational levels often get stuck in multi-step online workflows, while people with sensory or movement disabilities face innate operational limitations. If policies only launch online service entries without matching offline training and in-person service counters, such skill gaps will grow wider instead of being addressed (Bevilacqua et al., 2021).

Factors limiting usable opportunities come from the whole service environment, covering stable network signals, age-adapted device designs, volunteer guidance at community sites, and daily help from family members. Rural regions lack fixed spaces for regular digital training; older adults who live alone have no reliable contacts to solve payment malfunctions; people with disabilities need formal proxy permissions and offline alternative services. Online medical platforms can truly become usable only if they are matched with offline support systems tailored to the daily lives of older users (Kärnä et al., 2022).

Low willingness to participate mostly stems from lack of trust and poor self-confidence. Hard-to-read verification codes, hidden service fees, misleading links and bad past service experiences combine to create psychological resistance among older adults. Most older adults do not completely reject digital medical tools, but repeated operation failures and constant fear of fraud leave them with negative self-judgments, thinking they cannot learn such tools and will easily be scammed. Public behavioral policies can ease these mental barriers by simplifying operation steps, offering positive guidance, delivering anti-fraud education and setting up offline service alternatives (Małecka, 2021; Thaler & Sunstein, 2008).

Personal online safety hazards are an independent, vital layer in researching digital medical services. Operation mistakes, information leakage, confusing clinical data and unsmooth complaint channels all reduce older adults’ willingness to keep using online services (Percy Campbell et al., 2024). Institutional rules such as platform qualification standards and standardized complaint channels can offset these hidden dangers. The original three-part COM-B model does not cover personal online risks, yet such hazards heavily affect older adults’ long-term usage intentions, so this research takes them as a core analysis object (Scheibner et al., 2021). For this reason, the study expands the original three COM dimensions and adds risk supervision as a supplementary policy category under the M4 coding standard. We analyze whether various policy tools can reduce both institutional-level risks and individual safety threats via industry supervision, data privacy rules and user-oriented remedial services.

Policy design needs to match varied demands from different groups of older adults. Rural older adults put more weight on offline service stations; groups with little education need easy-to-read instructions and graphic guides; isolated elders require anti-fraud rules and trust-building activities; people with physical disabilities demand accessible interfaces and formal proxy mechanisms. All M1 to M4 coding work in this paper centers on four practical research questions: Can older adults complete operations independently? Can they obtain accessible services? Will they choose to participate voluntarily? Can they stay protected from risks? We avoid simple, surface-level classification of policy texts in all coding work.

### 2.2. Conceptualizing Digital Vulnerability and Policy-Built Digital Resilience for Older Adults

The digital vulnerability experienced by older adults arises from structural contradictions between personal conditions, the operational standards of online medical services, and available institutional support mechanisms (Xie et al., 2022). In practical research terms, this term describes lasting barriers that prevent older adults from stably using digital medical and eldercare tools. Such barriers root from insufficiencies in operational skills, service entry channels, usage motivation and safety protection. Specific real-world manifestations cover unsuccessful online bookings, trouble understanding chronic disease monitoring data, property losses caused by telecom fraud, digital platforms without offline backup services, and complete abandonment of online medical tools due to overly complicated steps (K. Kim et al., 2023). In this study, digital vulnerability is not understood as a rejection of digitalization itself. Rather, it emerges when digital channels become the sole or mandatory route for accessing health and eldercare services, while offline alternatives, human assistance, proxy mechanisms, and simplified communication remain insufficient. Under such conditions, digital service systems may form a discriminatory boundary: older adults with diminished digital capacities, sensory–physical impairments, low literacy, or insufficient family support gain formal access to service systems yet suffer practical exclusion from effective usage. This form of exclusion may trigger cascading tangible adverse risks: delayed chronic-disease monitoring resulting in aggravated physical conditions, continuous deterioration of informational health status, and direct financial losses induced by operational errors during digital health-related procedures. By contrast, non-discriminatory boundaries correspond to inclusive service ecosystems where online platforms coexist with offline service counters, human assistance and proxy service channels, enabling older adults to obtain equivalent health-related services through multiple alternative pathways. This research frames policy-built digital resilience as a comprehensive protective system formed by coordinated policy clauses. This concept cannot measure tangible changes in older adults’ daily behaviors or physical health, nor can it mirror how policies are carried out at grassroots levels. Instead, the term is used to judge whether complete four-layer supporting rules (covering ability cultivation, service access, motivation guidance and risk supervision) have been set out in policy documents; all analytical results in this research are only interpreted based on written policy materials (Estrela et al., 2023).

This study draws a clear boundary between written policy documents and their actual on-site execution. Any training-related clauses in official texts only indicate that authorities have acknowledged relevant challenges, instead of promising formal training activities. Descriptions of intelligent service platforms merely reflect written planning targets rather than fully functional online systems, and data protection rules cannot completely stop fraud or privacy leakage incidents. For this reason, every quantitative index and thematic analysis discussed later is only interpreted within the scope of collected policy records, without extending to offline practical situations.

### 2.3. Gaps in Existing Research

Current research on this topic can be split into two major analytical directions. One branch adopts health literacy measurement tools, technology acceptance frameworks and qualitative interview approaches to explore the internal factors that shape older adults’ choices of digital medical services (Oh et al., 2021). The second stream focuses on the supply-side arrangement of community-based care, institutional care, and smart older adult care systems (Murabito et al., 2024). While both strands acknowledge the importance of individual adaptation and institutional support, few studies have employed the COM-B framework as a bridging tool to connect micro-level behavioral patterns of older adults with macro-level policy texts (Lemos et al., 2024).

Research on older adult care policies has typically addressed community care, institutional operations, and medical–elderly integration as separate analytical domains, thereby fragmenting the complete service continuum upon which older adults depend (Cai et al., 2024; Tsai et al., 2024). To overcome this limitation, the present study constructs an integrated four-dimensional analytical framework that uniformly evaluates whether policy instruments adequately respond to older adults’ needs in terms of operational capability, service accessibility, usage willingness, and safety assurance (Hamiduzzaman et al., 2025).

The majority of COM-B applications remain confined to individual-level intervention experiments and questionnaire-based survey designs, with scarce usage in systematic policy-text coding that directly maps to older adults’ usage barriers (Jaana et al., 2025). This study does not seek to revise the original COM-B theoretical constructs; rather, it adapts the model to the specific context of China’s digital health governance and incorporates an M4 coding scheme to interpret institutional risk-prevention mechanisms embedded within policy documents (Liu et al., 2024).

### 2.4. Four-Dimensional Policy Framework and Research Questions

**Figure 1:**
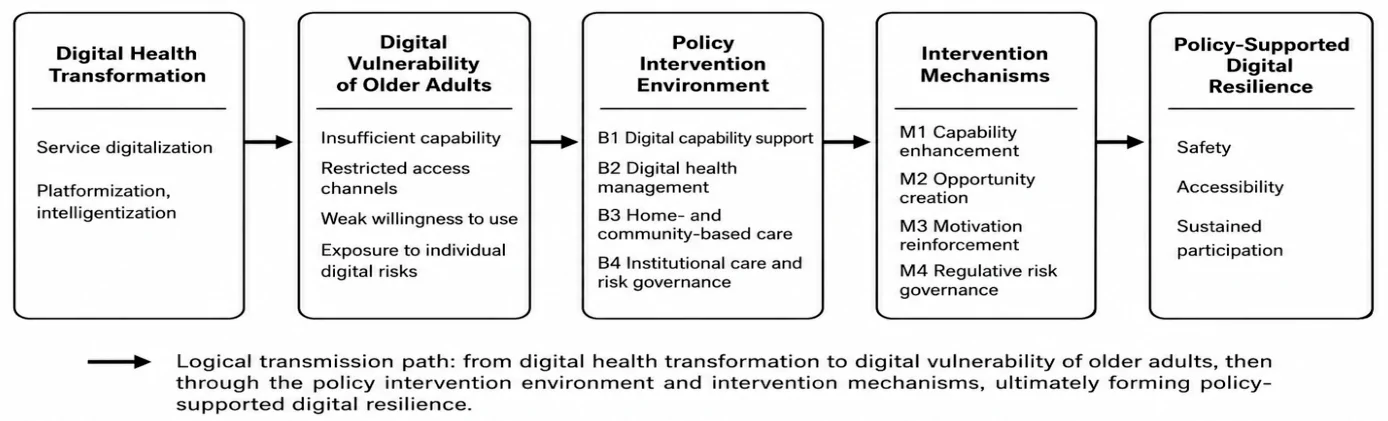
Older adults’ digital health behavioral barriers, policy intervention environment, and policy-supported digital resilience.

Addressing digital exclusion among older adults and fostering policy-supported digital resilience through institutional frameworks necessitates a comprehensive and coherent behavioral support policy system (Bertolazzi et al., 2024; Hettiarachchi Senarath et al., 2024). This study delineates policy provision into four primary dimensions: B1 (Digital Capacity and Age-Friendly Support), which responds to practical obstacles faced by older populations in terms of operation, information interpretation, and risk identification; B2 (Digital Health Management Support), which alleviates usage difficulties encountered in chronic disease online management and telemedicine participation; B3 (Home- and Community-Based Care Support), which establishes accessible offline assistance carriers in close proximity; and B4 (Institutional Care and Risk Governance), which safeguards the operational security of older adult care institutions, online service platforms, and personal data. Figure 1 visualizes the full logical relationship of this research framework.

All coded policy data point to a clear structural imbalance: most institutional resources are invested in physical service facilities and platform construction, whereas supporting policies designed to improve older adults’ digital skills are persistently insufficient. It is evident that policy resources are predominantly channeled toward online platforms, physical bed capacity, and industry oversight, whereas substantial gaps persist in the supporting chains for practical instruction, health information interpretation, and offline fallback mechanisms.

Based on the four-dimensional policy analysis framework and the core findings outlined above, this paper formulates three research questions:

**RQ1.**  *What are the evolutionary characteristics of China’s digital health policies for older adults from 2007 to 2025? From the sole perspective of policy texts, do relevant institutional adjustments adequately address the digital usage deficiencies and multifaceted safety risks encountered by older adults?*

**RQ2.**  *Has the focal emphasis of policy resources gradually shifted from conventional community- and institution-based hardware provision toward behavioral support initiatives directly targeting older populations—such as digital skills cultivation, autonomous health management, and individual digital risk protection?*

**RQ3.**  *Do current policy intervention instruments simultaneously cover the four key dimensions of capability enhancement, opportunity provision, motivation guidance, and risk regulation, thereby consolidating the institutional foundation for policy-supported digital resilience?*

## 3. Materials and Methods

### 3.1. Data Sources and Search Design

The retrieval vocabulary was designed to align with the four behavioral constraints of capability, opportunity, motivation, and individual digital risk among older adults, departing from generic search paradigms such as “older adult care services” or “digital economy.” Policy texts were extracted from the Peking University Legal Database (PKULAW), covering a timeframe from 1 January 2007, to 31 December 2025. The final database search and sample verification were conducted in July 2026, after the close of the 2025 calendar year. Accordingly, the policy records from 2025 constitute a complete annual retrieval window in this study, rather than a partial-year corpus. That said, the dataset is still confined to policy documents indexed in PKULAW that meet the screening criteria specified below. We set 2007 as the initial year of data collection mainly to ensure balanced longitudinal comparison and complete yearly policy samples. This time cutoff does not mean China’s eldercare and digital health-related policy releases only began in this year. However, this early-period pattern should not be interpreted as definitive evidence that no relevant local or informal support existed before 2018. It indicates that, within the PKULAW-indexed and legally binding corpus used in this study, policy-text attention to older adults’ digital adaptation was limited during the earlier years.

All keyword groups for document retrieval are designed to match the four-dimensional analytical framework defined above. B1 collects records about older adults’ digital literacy, age-adapted facility upgrades, hands-on smart device training and accessible information services. B2 includes texts focusing on integrated digital eldercare, remote medical consultations, chronic disease tracking, family doctor programs and combined medical–elderly service mechanisms. B3 extracts clauses for home care, community eldercare, family care beds, day care centers and older adult dining services. B4 gathers regulatory documents governing eldercare facility operation, safety oversight, star evaluation standards and digital management rules.

### 3.2. Text Screening Rules and Analytical Units

We set two screening standards to filter the full corpus. Only official policy texts with legal binding force and clear connections to digital health support for older adults were kept in the sample pool. We eliminated reprinted documents, policy explanatory files, draft consultation papers, project application forms, bidding materials, incomplete annexes, and generic eldercare documents that contain no specific digital health measures. Outdated policies that had been officially released yet later revoked were still retained in the dataset, regardless of their current legal status. The retained revoked or expired policies are used only for reconstructing the historical evolution of policy attention and institutional discourse. They are not treated as evidence of currently effective policy support. Therefore, when this study discusses current policy resilience or policy-supported digital resilience, the interpretation refers to the cumulative textual structure of the policy corpus rather than the legal effectiveness of every retained document at the time of analysis. This screening strategy also implies a methodological boundary. Because PKULAW mainly indexes formal policy documents with legal or administrative validity, regulatory and institution-oriented instruments are more likely to be retrieved than non-binding implementation notices, local training guidelines, or temporary community-level service arrangements. Therefore, the relatively low proportions of B1 and B2 should be interpreted as a pattern within legally binding and database-retrievable policy texts, rather than as a direct measure of all practical governmental inputs for older adults’ digital training and health-literacy support.

**Figure 2:**
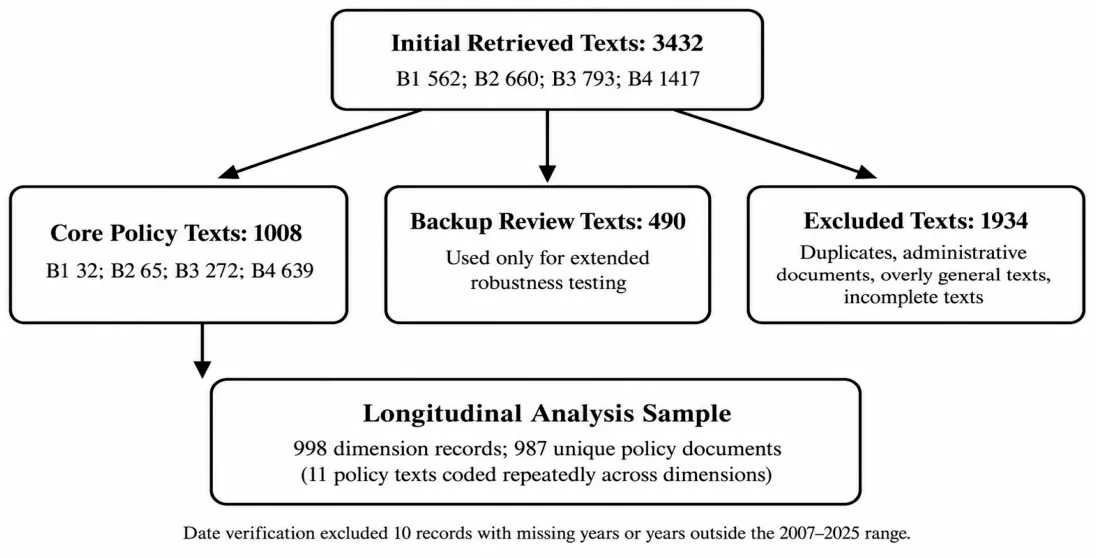
Policy text screening process and analytical sample.

To support time-series trend analysis and LDA topic mining, this research separates two distinct analysis units: dimension-labeled coded records and standalone policy files. Figure 2 displays the full document filtering workflow, while Table 1 lists sample volumes under each dimension category. After primary screening and chronological verification, we collected a total of 1008 core policy records. From this pool, 998 multi-tagged dimension records were used to calculate yearly structural proportions of the four B frameworks (B1 to B4). After deduplication, 987 independent policy texts were retained for topic modeling and intervention mechanism coding. An additional 490 alternative texts were included solely in an expanded sample for robustness testing and were not incorporated into the primary empirical analysis. In Table 1 and Figure 2, “Core Pool” refers to the 1008 policy records retained after primary screening. “Annual Dimension Records” refers to the 998 B1–B4 dimension-coded records used in Section 4 for yearly structural analysis after excluding 10 records with missing or unverifiable year information. “Independent Policy Texts” refers to the 987 deduplicated documents used for LDA topic modeling and M1–M4 mechanism coding.

### 3.3. Behavior-Oriented Coding and Review Procedure

The B1–B4 and M1–M4 coding schemes were applied to the identical full policy corpus but serve distinct analytical purposes. The B1–B4 dimension coding supports multi-tagging: one policy containing multiple digital health related clauses will generate multiple independent coded records, leading to a total of 998 dimension records for annual longitudinal analysis. In contrast, M1–M4 mechanism coding extracts one dominant intervention type for each deduplicated unique policy document, forming 987 independent documents for LDA and mechanism proportion calculation. The two systems are theoretically connected through the extended COM-B model, but this study does not implement a full crossed 4 × 4 statistical matrix for cross-analysis.

Documents that merely mention older adult care in general terms without incorporating substantive digital support provisions were not classified under B1 (Digital Capacity Support). Policy content pertaining to the establishment of community service stations, in-person consultation services, and volunteer assistance programs was categorized under opportunity-creation measures. Provisions addressing anti-fraud awareness campaigns, practical device operation training, and health data interpretation guidance were assigned to either capability-enhancement or motivation-oriented pathways. Following this coding scheme, policy text statements were transformed into quantifiable behavioral support variables, with complete definitions of each coding category presented in Table 2.

To reduce subjective bias during manual text coding, we randomly selected 20% of all policy records (n = 202) for independent secondary coding by a separate researcher. We separately calculated pairwise percentage agreement and Cohen’s kappa to quantify inter-coder reliability for the two coding frameworks. For B1–B4 dimension coding, the pairwise percentage agreement reached 93.1%, with a Cohen’s kappa coefficient of 0.86 (indicating almost perfect agreement); for M1–M4 intervention mechanism coding, the pairwise percentage agreement was 81.2%, accompanied by a Cohen’s kappa coefficient of 0.65 (indicating substantial agreement). All coding disagreements were resolved through joint review of the coding dictionary and original policy texts.

### 3.4. LDA Topic Model

We use LDA topic modeling to trace thematic shifts across the full corpus of independent policy documents and to examine whether governance priorities moved from institutional oversight toward support for older adults’ digital adaptation and online risk mitigation (Blei et al., 2003). This study used 987 independent policy documents as the analytical corpus. During the preprocessing stage, webpage links, database citation codes, document closing remarks, duplicate titles, and meaningless symbols were removed; high-frequency stop words were filtered out; and 2- to 4-character n-grams were extracted to capture specialized policy terminology. In this analysis, Chinese policy texts were vectorized using a Chinese-character n-gram strategy. Specifically, after removing PKULAW metadata, webpage links, administrative numbering expressions, boilerplate closing formulas, and non-Chinese character noise, CountVectorizer in scikit-learn version 1.3.2 was used to extract 2- to 4-character Chinese n-grams. This character n-gram strategy was used instead of a word-segmentation engine such as Jieba or HanLP because many policy expressions, such as “smart eldercare,” “integrated medical and elderly care,” “age-friendly transformation,” “offline service counters,” “proxy services,” “telemedicine,” and “anti-fraud education”, are short fixed phrases that may be fragmented by ordinary word-segmentation tools. Terms appearing in fewer than 10 documents or in more than 85% of documents were filtered out, and the maximum number of features was set to 1000 to reduce sparse noise.

To reduce arbitrariness in topic-number selection, the candidate topic range was expanded from K = 5–8 to K = 5–20. For each candidate K value, perplexity, UMass coherence, an NPMI-based coherence proxy, and manual interpretability were jointly considered. LDA topic modeling was implemented using LatentDirichletAllocation in scikit-learn version 1.3.2. Symmetric Dirichlet priors were adopted for both the document–topic distribution and the topic–word distribution, with α = 1/K and β = 1/K for each candidate topic number. For the final K = 7 model, α = β = 1/7 ≈ 0.1429. The full expanded comparison from K = 5 to K = 20 is reported in Table A1 in Appendix A. Although perplexity generally decreased as K increased, higher K values produced increasingly fragmented and overlapping topics. Therefore, K = 7 was retained as the final model because it achieved the best balance between statistical performance and substantive policy interpretability (Table 3).

It is worth emphasizing that lower perplexity only reflects improved model-data statistical fit. Excessively high K values lead to fragmented themes that lose practical meaning for policy-oriented analysis. Our final choice of K = 7 prioritizes the trade-off between statistical metrics and real-world thematic interpretability tailored for Chinese policy texts, with full parameter comparisons provided in Appendix A.

### 3.5. DAPOI, BIDI, and Research Boundaries

After extracting the full thematic distribution from the LDA modeling results, we developed two quantitative indicators to quantify how policy focus varies among the four behavioral constraint dimensions defined in this research.

The Digital Adaptation Policy Orientation Index (DAPOI) quantifies the share of policy record content addressing older adults’ individual digital limitations, such as difficulties in device operation, health information interpretation, and autonomous participation in digital health services. Higher values indicate stronger policy attention to older adults’ individual-level digital adaptation.

A higher index value indicates that policy content places greater emphasis on addressing individual-level shortcomings among older adults—such as digital proficiency, health information discernment, and self-directed engagement in health management. The DAPOI is calculated as the proportion of coded records in dimensions B1 and B2 relative to the total number of coded records across all four dimensions, using the following formula:

(1) $$\begin{array}{c} DAPOI_{t}=\frac{B1_{t}+B2_{t}}{B1_{t}+B2_{t}+B3_{t}+B4_{t}} \end{array}$$

In Equation (1), $B_{1t}$, $B_{2t}$, $B_{3t}$, $B_{4t}$ denote the numbers of coded records for dimensions *B1*, *B2*, *B3*, and *B4* in year t, respectively. The Behavioral Intervention Diversity Index (BIDI) identifies the structural characteristics of policy intervention patterns. It is used to assess whether the governance logic shifts from single supply or one-way regulation toward multidimensional collaborative intervention involving capability, opportunity, motivation, and risk governance (Simpson, 1949). A higher value indicates a more balanced distribution of intervention tools in policy texts, which can cover multiple support conditions for older adults’ digital health behaviors. Let $p_{m,t}$ denote the proportion of the m-th intervention mechanism in year t. The denominator 0.75 represents the theoretical maximum value of Simpson’s diversity transformation under four equally distributed intervention mechanisms. When M1, M2, M3, and M4 are perfectly balanced, each mechanism accounts for 0.25 of all intervention codes. Thus, the maximum diversity value is calculated as 1 − 4 × (0.25)^2^ = 0.75. Dividing by 0.75 normalizes BIDI to the interval from 0 to 1, making annual values comparable across the study period.

For the DAPOI metric, no year within our coded dataset produces a zero denominator. Even so, several early years contain extremely small total coded policy records, which may lead to high variance for annual proportional indicators. We deliberately avoid numerical smoothing operations, as smoothing would alter raw policy-text frequency information and distort original empirical evidence. Therefore, DAPOI figures from years with sparse policy records are interpreted merely as directional descriptive signals rather than precise quantitative point estimates. Furthermore, parametric confidence intervals are not calculated in this research. Our dataset originates from manual clause-level qualitative coding of policy documents instead of random sampling, which fails to satisfy core statistical prerequisites required for parametric confidence-interval computation.

(2) $$\begin{array}{c} BIDI_{t}=\frac{1-\sum_{m=1}^{4}p_{m,t}^{2}}{0.75} \end{array}$$

In Equation (2), $p_{m,t}$ refers to the proportion of the *m-th* intervention mechanism (M1–M4) among all intervention codes in year *t*, and *m* corresponds to the four intervention mechanisms. Consistent with the boundary of the LDA model, the two indices must be interpreted with caution. DAPOI only reflects the relative policy-text attention to older adults’ digital adaptation issues, while BIDI only reflects the textual balance among intervention mechanisms. Neither index measures policy budgets, implementation effects, coverage of digital training, actual usability of digital service platforms, or substantive changes in older adults’ digital health behavior, physical health, or real-world well-being. Because DAPOI and BIDI are sample-internal relative indicators constructed in this study, no absolute high-low thresholds are set. The interpretation is based mainly on annual changes, stage comparison, and mutual corroboration with the four-dimensional and intervention-mechanism coding results. It should also be noted that both DAPOI and BIDI are unweighted text-frequency indicators. Each retained policy document or coded record is treated as one analytical unit, regardless of issuing authority, legal hierarchy, budget size, implementation duration, or geographic coverage. Therefore, these indices do not measure policy strength, legal force, fiscal input, or practical implementation scope. They are designed to capture the relative textual orientation and intervention diversity within the collected policy corpus.

### 3.6. Robustness Design

Based on the measurements of DAPOI, BIDI, and the proportional distribution across all four dimensions, this study identifies a structural pattern characterized by a heavy concentration of policy resources on the supply side and a persistent insufficiency in individual capacity-building support. To rule out potential interference arising from sample screening criteria, this study incorporates 490 alternative texts to construct an expanded sample and compares the four-dimensional coding distributions between the two groups. If both samples consistently exhibit higher proportions in B3 and B4 and lower proportions in B1 and B2, this would confirm that the observed resource allocation tendency is stable and not a spurious result attributable to differences in sample composition.

## 4. Results

### 4.1. Overall Time-Series Distribution of B1–B4 Policy Records

**Figure 3:**
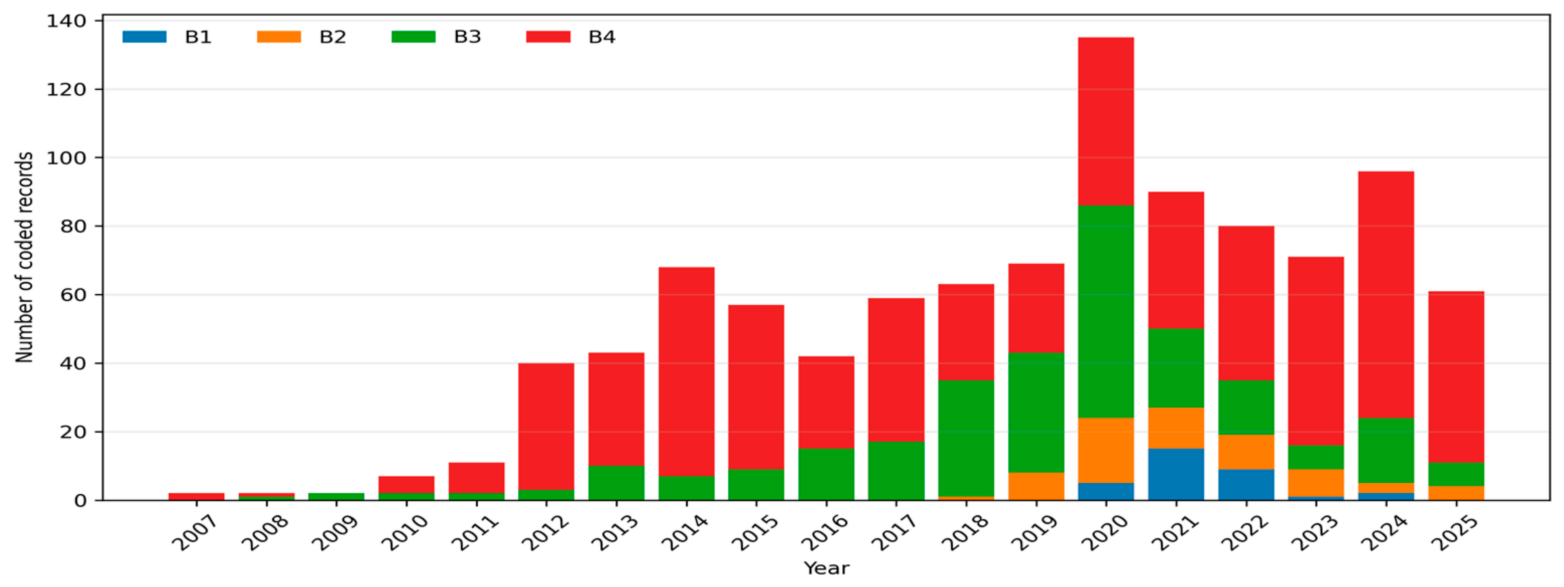
Annual evolution of B1–B4 policy support dimensions.

Figure 3 tracks yearly changes in the volume of coded records for the four policy dimensions B1 to B4 from 2007 to 2025. Between 2007 and 2017, policy content centered mostly on institutional access rules, operation norms and offline care facilities. Most policy clauses focused on building and running digital medical platforms, with very few rules targeting older adults’ independent device operation and self-managed health data analysis. Longitudinal statistics show that B3 and B4 took up the vast majority of policy records in this timeframe, while materials linked to B1 and B2 appeared only rarely. This pattern intuitively reflects a policy supply structure that prioritized the development of hardware platforms and service institutions, without commensurate progress in supporting older adults’ digital literacy and self-directed health management. Tasks such as practical digital training, anti-fraud education, and health-information interpretation guidance for older adults had not yet been institutionalized as routine policy arrangements.

Around 2020, the difficulties faced by older adults in using smart technologies gradually emerged as a prominent policy issue. As observed in the trend lines of Figure 3, the volume of coded records in dimension B1 increased markedly between 2020 and 2022, corresponding to policy responses targeting practical usage barriers including QR-code verification, online appointment registration, digital payment, and smart device operation. However, within the PKULAW-indexed corpus, the B1 counts declined to some extent after 2023, suggesting that policy-text attention to older adults’ digital operational capacity retains a strong element of short-term emergency response, and has yet to be transformed into a durable, long-term institutional arrangement that permeates the entire spectrum of digital health services for older adults. It should be noted that annual coded policy counts remain very limited throughout 2007–2017. Annual ratios in this early phase need cautious interpretation: these values mainly reflect low policy attention on textual records, and shall not be treated as precise quantitative benchmarks for direct year-by-year comparison against observations after 2018. Major research conclusions rely more on three-stage aggregated group comparison rather than fluctuations of individual early-year data points.

### 4.2. Four-Dimensional Structure: Supply-Side Policies Dominated, While Individual Capability Support Was Insufficient

This study calculates the number and proportion of core coded records for the four dimensions to present the static distribution of policy attention. As shown in Table 4, B1 digital capability and age-friendly support and B2 digital health management support, both of which focus on individual empowerment for older adults, have limited numbers of records and together account for less than one tenth of the total. In contrast, B3 home- and Community-Based Care Support and B4 institutional care and risk governance, which focus on external service scenarios and industry governance, together account for more than 90%.

This distribution indicates that past policies have placed greater emphasis on service site construction, facility provision, institutional operational standards, and industry risk control, while long-term cultivation of older adults’ digital adaptation capabilities has not received sufficient attention. In reality, many older adults cannot independently complete online procedures or payments and need assistance to access digital health services. The support measures most urgently needed by these groups are precisely those concentrated in the lowest-proportion B1 and B2 dimensions.

Such uneven proportional distributions do not stem from random sample gaps; they reflect long-term skewed allocation of policy priorities. Dimension B4 accounts for the largest share, reflecting sustained policy emphasis on risk governance at the institutional level. Dimension B3 follows closely, indicating continued progress in the provision of inclusive home- and community-based care services. However, the persistently low proportion of dimension B1 reveals a long-standing deficiency in policy resources allocated to individual empowerment—including operational guidance, health information interpretation, and online risk identification for older adults. While policies have consistently expanded the coverage scenarios of digital health services for older adults, the corresponding individual-level support necessary for older adults to use these services independently has remained chronically inadequate.

### 4.3. DAPOI: Digital Adaptation Issues Rose Temporarily but Were Not Institutionalized Stably

The DAPOI captures time-series changes in policy attention toward older adults’ individual digital capability and autonomous health management. The results show that the index remained close to zero before 2018 within the PKULAW-indexed corpus. This low early value may reflect both the later emergence of digital health and age-friendly digital governance as salient policy issues and the possibility that early local, informal, or non-binding support measures were not fully captured by the database. With the rise of smart health and eldercare and age-friendly digital policy issues, the index gradually increased from 2019 to 2020 and reached a stage peak of 0.300 in 2021, after which it declined. Compared with the static dimensional structure shown in Section 4.2, DAPOI does not represent a fixed allocation pattern of policy resources. Instead, it reveals a clear time lag in support for individual digital adaptation. Readers should keep in mind that DAPOI estimates before 2018 are generated from sparsely coded annual records; these outputs serve as directional descriptive signals instead of rigorous precise quantitative estimates.

### 4.4. Stage-Based Evolution of LDA Policy Topics

The LDA topic model was used to mine policy topics from all policy texts. Seven semantically stable topics were extracted. The core terms, governance direction, and correspondence with older adults’ digital health needs are summarized in Table 5.

As reflected in the thematic connotations presented in Table 5, current policy topics are predominantly oriented toward macro-level supply-side dimensions—including institutional operations, infrastructure development, community-based service sites, and industry oversight—while independent topics dedicated specifically to individual-level adaptive needs, such as digital operation training, plain-language health information interpretation, and online risk discernment for older adults, remain relatively scarce.

**Figure 4:**
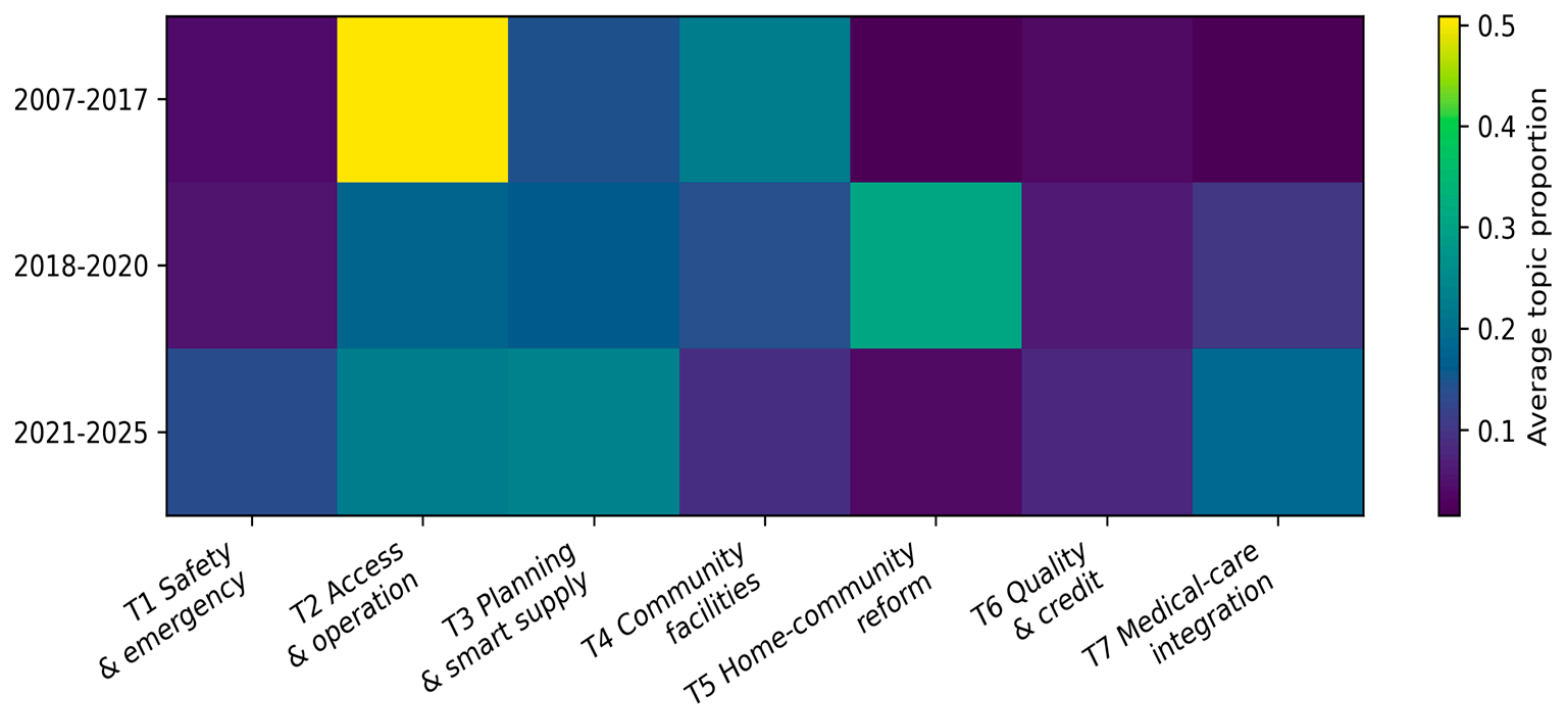
Stage-based distribution of LDA topics.

To clearly present the dynamic evolution of policy topics from 2007 to 2025, the entire study period was divided into three phases: 2007–2017, 2018–2020, and 2021–2025. The average proportional share of each topic within each phase was calculated, and the resulting stage-wise distributions are visualized in the heatmap presented in Figure 4. From 2007 to 2017, policy formulation mainly targeted standardized management for eldercare facilities. Topic T2 covering institutional entry and daily operation occupied far more policy space than other thematic types. Meanwhile, supportive measures including digital skill training, online privacy safeguards and authorized surrogate services had not yet received enough policy attention at this stage. In the 2018–2020 period, home- and community-based care policies expanded steadily, with topics T4 (community care) and T5 (home-based care pilots) exhibiting notable increases, as grassroots service settings began to emerge as potential vehicles for digital assistance. From 2021 to 2025, the policy scope broadened further, with multiple thematic areas—including safety emergency response, smart platforms, and medical–elderly health integration—developing in parallel. Nevertheless, content specifically addressing the cultivation of digital capabilities among individual older adults remained outside the mainstream. Taken together, the long-term trends revealed by the heatmap indicate that over more than two decades, policy construction has consistently prioritized institutional regulation and hardware provision, while complementary support targeting older adults’ digital usage deficiencies has been persistently under-supplied.

### 4.5. M1-M4 Mechanisms: Strong Risk Governance and Weak Capability Enhancement

Based on the coding results, the distribution of the four policy intervention mechanisms was calculated. The scale and proportion of different intervention tools are shown in Table 6.

The proportional distribution of the various mechanisms directly reflects a pronounced structural mismatch in policy instruments. Capability-enhancing (M1) entries account for only 7.80%, making them the smallest among the four intervention types. The support that older adults require to adapt to digital health services consists not primarily of one-off awareness campaigns, but rather of sustained hands-on guidance, in-person accompaniment, health data interpretation, and risk identification training. Rural older adult populations depend on regular grassroots-level tutoring; less-educated older adults need simplified instructional approaches; socially isolated older adults require accessible and immediate help channels; and disabled older adults rely on caregiver proxy services and accessible operation solutions. These types of individual-oriented support cannot be spontaneously generated through institutional regulatory frameworks alone.

Regulative risk governance (M4) accounts for 58.36%, indicating a long-standing policy preference for building risk prevention and control systems. Market access criteria, operational standards, supervisory inspections, credit management, emergency safeguards, and data privacy regulations effectively mitigate macro-level hazards such as institutional operational disorder, service interruptions, and information breaches. However, institution-oriented regulatory constraints do not directly translate into digital safety assurances for older adult end users. If assessment standards focus solely on institutional compliance documentation while neglecting user-oriented anti-fraud alerts, comprehensible privacy consent explanations, error-blocking mechanisms, and complaint and exit channels, a paradoxical situation readily emerges—where platforms are compliant and secure by regulatory metrics, yet older adults encounter substantial difficulties in actual usage.

**Figure 5:**
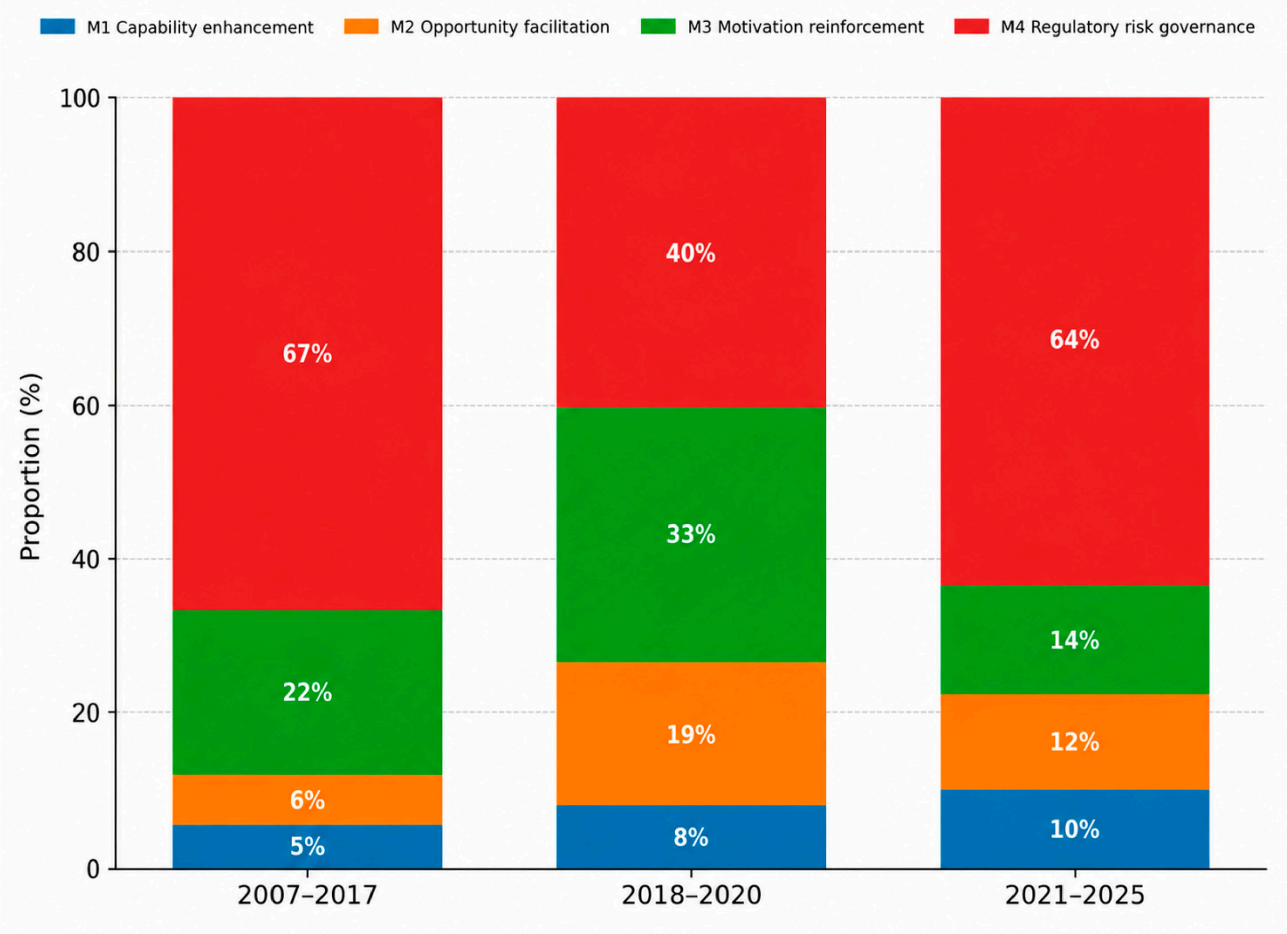
Stage-based structure of dominant policy intervention mechanisms.

The dynamic evolution of the four intervention mechanisms across different periods is illustrated in Figure 5.

### 4.6. DAPOI and BIDI: Policy Orientation and Tool Combination Did Not Form a Stable Closed Loop

**Figure 6:**
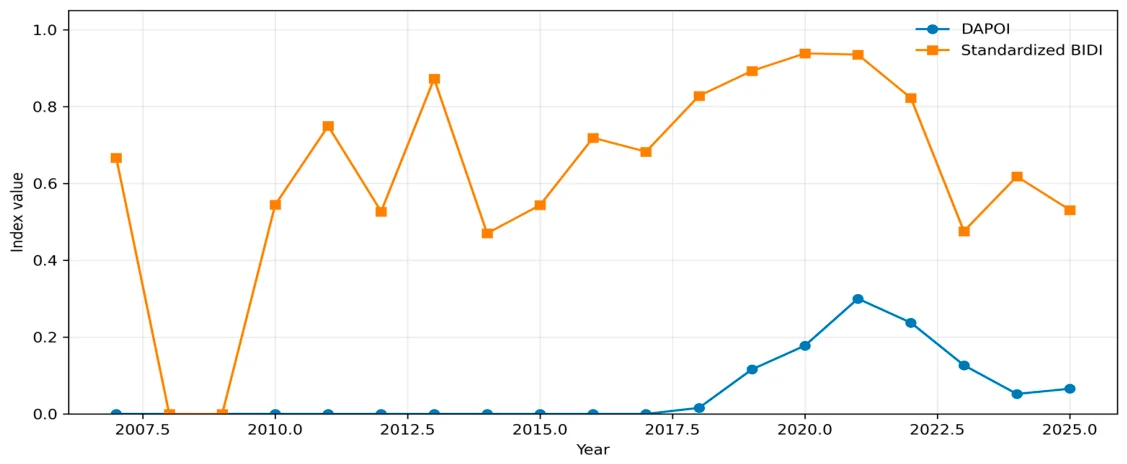
Annual changes in DAPOI and standardized BIDI.

The sustained use of digital health services by older adults relies on the synergistic support of four essential elements: capability, opportunity, motivation, and risk governance. The temporal evolution of the policy intervention mix and the DAPOI is presented in Figure 6. From 2018 to 2021, the BIDI exhibited a continuous upward trend, indicating that policy intervention approaches gradually moved beyond a singular regulatory model, with a steady enrichment of diversified tool combinations. In 2021, both the DAPOI and BIDI reached relatively high levels simultaneously, suggesting that policy texts during this phase achieved a comparatively balanced configuration across the four instrument categories—individual capability, usage opportunity, participation motivation, and risk governance. Compared to a unidimensional supply-focused approach centered on platform development, this diversified combination model is more conducive, at the textual level, to establishing a policy environment that concurrently ensures user-friendliness, accessible help channels, and risk protection. After 2022, both indices declined in parallel within the coded corpus, suggesting a reorientation of policy emphasis toward institutional informatization supply and industry oversight, accompanied by a weakening of sustained support for older adults’ digital adaptive capacities.

### 4.7. Robustness Test with the Extended Sample: Structural Imbalance Was Not Driven by Screening Scope

To verify that the distribution of dimensions was not disturbed by the sample screening rules, this study included 490 backup texts to build an extended sample and compared the B1–B4 proportions between the two sample scopes. The results are shown in Table 7.

The dimensional distributions of the two samples are highly consistent, with B3 and B4 consistently accounting for the vast majority of coded records, while the proportions of B1 and B2 remain persistently low. Even after expanding the text screening scope, policy content pertaining to hands-on training for older adults, health information interpretation, anti-fraud education, and manual fallback support remains under-supplied, indicating that the observed allocation pattern—where policy resources are disproportionately directed toward hardware services and institutional oversight—is robust. This robustness check is limited to comparisons of the four-dimensional (B1–B4) distributions. The robustness of the LDA topic evolution, intervention mechanism structures, and the temporal trajectories of the DAPOI and BIDI indices awaits further validation through expanded samples in subsequent research.

## 5. Discussion

### 5.1. Analytical Perspective and Theoretical Interpretation

The accessibility of digital health services is tightly tied to older adults’ physical and mental wellbeing. Existing research on seniors’ digital difficulties is split into two isolated research streams: one adopts questionnaires and interviews to explore micro-level usage barriers, while the other focuses on the supply layout of community and institutional eldercare systems. Few prior studies systematically link older adults’ digital vulnerability to top-down policy design, forming a notable research gap. To fill this gap, this study adapts the COM-B behavioral model for quantitative policy text analysis. Based on the 2007–2025 policy corpus, we map seniors’ constraints in capability, opportunity, motivation and risk exposure to corresponding coded policy provisions. This integrated analytical framework addresses all three research questions and provides a behavioral public policy perspective to interpret policy-supported digital resilience.

Overall results indicate that China’s digital health policies for the elderly have expanded gradually, shifting from single institutional regulatory rules to multi-dimensional governance covering community care, medical-elderly integration, smart platform construction and risk supervision. Nevertheless, clauses targeting seniors’ independent digital operation and easy-to-understand health data interpretation remain scarce within our coded policy corpus. This textual imbalance demonstrates that institutional supervision and hardware construction alone cannot cultivate complete digital resilience; policies also need to tackle personalized risks such as operational errors, false medical information, confusing privacy clauses and telecom fraud.

By integrating four-dimensional coding, M1–M4 mechanism classification, LDA thematic mining and the self-developed DAPOI and BIDI indices, this study helps bridge the long-standing analytical divide between individual digital hardships and macro institutional policy supply. It avoids the disjointed logic seen in most previous literature that separates micro user barriers from macro policy supply analysis.

### 5.2. Uneven Allocation of Policy Resources: Textual Patterns and Plausible Institutional Interpretations

The uneven textual distribution observed in the coded corpus should be interpreted as a pattern shaped by both substantive policy priorities and methodological artifacts stemming from retrieval and screening rules. On the one hand, the dominance of B3 and B4 aligns with the institutional governance preference reflected in formal regulatory documents indexed by PKULAW. On the other hand, B1-type digital literacy training and B2-type health management guidance are mostly released in non-binding local notices and grassroots implementation guidelines that are not fully collected in this legal database, so their low share in the coded corpus cannot be equated to insufficient overall government investment. Hardware indicators such as eldercare bed quantities, community service stations and intelligent equipment deployment are easy to quantify in routine administrative assessment, while skill-building training generates long-cycle benefits with few short-term measurable outputs, which may reduce sustained local investment incentives. These institutional characteristics offer a plausible deductive interpretation of our coding results derived from prior literature, yet such reasoning is not direct causal evidence extracted exclusively from the PKULAW policy texts analyzed in this study (Wei et al., 2026).

In terms of task division between government branches, institutional supervision and community facility construction belong to regular work of civil affairs and health bureaus, which receive steady fiscal support and fixed project arrangements. In contrast, improving older adults’ digital literacy demands joint work across multiple departments without a clear leading authority, which makes long-term stable policy input hard to maintain.

Outdated administrative thinking also widens such resource gaps. Previous policy planning generally assumed older adults could independently master digital services, ignoring major usage difficulties experienced by rural residents, the oldest elderly, people living alone and groups with physical disabilities. Governments usually shift the burden of digital learning and risk recognition to older adults and their relatives. This arrangement may help explain a long-standing textual pattern: policy documents continue to emphasize hardware facilities, while targeted personalized digital support measures remain limited (Dai et al., 2026)

### 5.3. Risk Stratification and Digital Resilience Cultivation: Supply–Demand Mismatch Between Institutional Regulation and Individual Protection

Policy tools show obvious structural imbalance: supervision and regulation clauses take up the largest share of policy texts, while supporting rules that help older adults improve digital skills stay inadequate for years. The changing DAPOI values confirm this observation, which corresponds to the second research question (RQ2). Rules governing market entry, daily supervision and data protection can curb systemic risks like institutional irregularities and information leaks, yet such policies cannot resolve personalized safety troubles that older adults face in real use scenarios (DeLange Martinez et al., 2024). Such limitations exist because most regulatory policies restrict platform operators and service suppliers instead of creating targeted guidance to fix older adults’ difficulties in operating devices and understanding medical content. No transitional supporting measures are set up to bridge the gap between institutional compliance standards and ordinary older adults’ online safety, such as one-on-one skill coaching, easy-to-read risk reminders and offline manual service channels. To tackle personal risks like wrong operations, obscure privacy contracts, false medical information and telecom scams, authorities need to roll out more capacity-building policies, such as recurring hands-on training, simplified health explanations and long-term anti-fraud publicity (Chen et al., 2024).

Such discriminatory exclusion may trigger cascading adverse consequences, including delayed chronic-disease surveillance, deteriorated informational health status, and economic losses arising from user operational mistakes.

From the perspective of public-health information modeling, offline alternatives and human-assisted proxy mechanisms should not merely be regarded as temporary welfare-oriented supplements. Instead, they constitute essential societal legal reasonable-accommodation measures and core institutional safeguards, preventing mandatory digital workflows from excluding older adults suffering from visual impairment, mobility restrictions, low literacy or inadequate family care resources. In this regard, opportunity-facilitating M2 measures including offline service counters, manual support, simplified workflows and proxy authorization transform digital-health systems from formally available platforms into genuinely accessible public services.

We analyze year-by-year changes in the DAPOI and BIDI to track policy shifts. Between 2018 and 2021, policy design gradually adopted multi-angle collaborative governance, distributing resources evenly across capacity improvement, service access, motivation stimulation and risk prevention. After 2022, policy focus shifted back to building institutional hardware and industrial supervision, and this balanced multi-tool governance mode could not be maintained continuously. Interpreted under the COM-B framework, the whole set of policy resources leans heavily toward external service facilities and top-down risk management, whereas supporting systems meant to offset older adults’ digital operation difficulties are far from sufficient. This result matches the analysis targeting RQ3. This suggests at the textual level that existing policy documents have not yet formed a complete set of external safeguards covering all four dimensions required for older adults to access digital medical services safely and smoothly.

### 5.4. Policy Optimization Paths and Practical Implications for Differentiated Older Adult Groups

The results derived from the preceding analysis demonstrate lasting unevenness in policy resource distribution: few institutional inputs target measures that help older adults adapt to digital medical services. In addition, various subgroups of older adults face distinct difficulties and safety threats when using online health tools. One-size-fits-all policy arrangements therefore cannot satisfy their differentiated demands. Policy design thus requires a stratified and targeted intervention approach. Drawing upon the real-world difficulties faced by four distinct older adult subgroups—those with low educational attainment and advanced age, rural residents, solitary older adults, and disabled or semi-disabled individuals—this study maps the corresponding risk prevention objectives and tailored support pathways for each group. The detailed stratified governance framework is presented in Table 8. It should be noted that this study did not conduct subgroup-specific statistical analyses of policy texts by urban–rural residence, age cohort, or functional status. The stratified pathways outlined in the table are derived deductively, grounded in the COM-B behavioral barriers framework, the overall conclusions regarding policy supply imbalances, and existing literature on older adults’ digital vulnerability.

As shown in the stratified framework presented in the table, the sources of digital vulnerability differ markedly across the four older adult subgroups, and the corresponding intervention measures are not mutually substitutable. For low-education and advanced-age groups, priority should be given to cognitive adaptation and hands-on operational training; for rural older adults, the focus lies in supplementing grassroots offline support infrastructure; for solitary older adults, the core need is to strengthen risk early-warning systems and help-seeking channels; and for disabled older adults, rigid human-replacement services and standardized proxy arrangements are indispensable. The central logic of stratified governance is to move beyond the conventional policy approach that prioritizes hardware supply while neglecting individual-level adaptation, and instead align targeted intervention instruments with the specific deficiencies of each group, thereby filling the gaps in stratified support within the current policy system.

In conjunction with the overarching conclusion of policy supply imbalance drawn throughout this study, the evaluation and assessment framework for digital health governance also requires simultaneous optimization. Current policy assessment frameworks largely rely on hardware-related benchmarks, such as the quantity of intelligent platforms, available eldercare beds and community service coverage. Few indicators capture older adults’ real usage experience (Qiu et al., 2024). Subsequent evaluation systems for policy execution need to add behavior-focused measurement criteria. Examples include the success rate of older adults completing online medical procedures, the availability of offline alternative service windows, simplified and easy-to-understand privacy agreements, and the frequency of continuous digital skill training. Permanent digital guidance hubs can be set up within community health centers and eldercare venues. Meanwhile, authorities need to build regular, tailored support schemes for disadvantaged groups, namely the oldest-old, people living alone and older adults with disabilities. Such arrangements can effectively fill the shortage of policy resources designed to enhance individuals’ digital capabilities.

## 6. Conclusions

### 6.1. Main Findings

This paper analyzes policy documents on older adults’ digital health issued between 2007 and 2025. The empirical analysis adopts a four-dimensional supply framework, intervention category classification, LDA thematic modeling, alongside two quantitative indicators: DAPOI and BIDI. Targeting the three research questions (RQ1, RQ2 and RQ3), the main results are summarized as follows.

Evidence from LDA thematic outputs, time-series indicator computation, and extended-sample robustness tests consistently shows that, within national and macro-level policy directives indexed by PKULAW, policy priorities have focused mainly on institutional access rules and community hardware construction over a long period. By contrast, clauses designed to improve older adults’ digital skills only occupy a minor proportion of all policy content. Only during the 2018–2021 period did policies briefly tilt toward individual digital adaptation and diversified interventions, a trend that gradually receded after 2022. Across different text-screening criteria, the structural pattern—where policy resources are heavily concentrated in community and institutional sectors while individual-level adaptive support remains insufficient—remains consistently stable. Overall, although the scope of policy coverage has steadily expanded, the resource allocation imbalance across the four dimensions is pronounced. The age-friendly support policies introduced around 2020 appear to represent only short-term adjustments rather than the establishment of lasting individual empowerment mechanisms.

In terms of the proportional composition of the four policy intervention types, the current governance model relies heavily on industry regulatory constraints, while capacity-building policies capable of enhancing older adults’ digital literacy and mitigating individual online risks are markedly under-represented, with multidimensional coordinated intervention having been achieved only briefly and temporarily. From the COM-B perspective, the existing policy texts have yet to establish a complete set of external support conditions that simultaneously address capability enhancement, opportunity provision, motivational guidance, and risk protection. Current regulatory policies can only mitigate systemic risks at the institutional level, but remain inadequate in resolving individualized usage challenges—such as operational errors and telecommunications fraud—encountered by older adults.

Current policy evaluations predominantly center on hardware infrastructure metrics, lacking assessment criteria that capture older adults’ actual user experiences. Future governance should adopt a stratified and targeted support approach, incorporating practical training, offline fallback services, and related performance indicators into the evaluation framework, so as to fill the supply gaps in policies oriented toward older adults’ digital adaptation.

### 6.2. Research Limitations and Policy Implications

#### 6.2.1. Policy Implications

Existing digital health policy evaluations largely focus on platform- and institution-level hardware indicators. Future assessment frameworks should shift toward older adults’ real-world user experiences. Local governments can establish routine digital assistance stations at community service sites, primary healthcare facilities, and older adult care venues, offering ongoing hands-on training in appointment registration, payment processing, chronic disease management, privacy protection, and anti-fraud education. Dedicated in-person service channels should be opened for the oldest-old, solitary, and disabled older adults, while supporting mechanisms—including authorized proxy arrangements and online complaint procedures—should be refined to address the current deficiencies in individual-level digital adaptation policies.

#### 6.2.2. Research Limitations and Future Directions

This study relies exclusively on policy texts as the sole analytical material, and therefore can only characterize resource allocation patterns at the textual level. It is unable to capture the actual service scenarios following policy implementation, nor can it verify whether institutional provisions effectively translate into tangible improvements in older adults’ digital operational skills or health-related behaviors.

All policy samples in this research are extracted exclusively from the Peking University Legal Database (PKULAW). Therefore, local supplementary normative documents, grassroots implementation guidelines and project implementation records may not be fully covered. In particular, the scarcity of records in the earliest years may partly reflect incomplete digitization or database coverage of local and non-binding documents, rather than a complete absence of relevant policy activity. Therefore, early-stage low-frequency results should be interpreted as limited textual visibility within the selected database, not as definitive evidence of total policy absence. We set 2007 as the initial year mainly to ensure balanced sample distribution across different time periods. Subsequent research could expand the scope of local policy data sources to enrich the textual sample. Additionally, this study conducted cross-validation on only 20% of the coded sample; future quantitative text-analysis studies of a similar nature may employ full-sample double-blind coding, with standardized reporting of inter-coder reliability, to enhance analytical rigor.

Future research may introduce weighted policy-text indicators by considering issuing authority, document type, legal effectiveness, implementation scope, and fiscal commitment, thereby distinguishing national-level foundational laws from local implementation notices and project-based guidelines. In the longer term, follow-up research could integrate multi-source data—including questionnaire surveys, in-depth interviews, platform operational data, and grassroots intervention experiments—to construct a mixed-methods analytical framework that combines macro-level policy environments, older adults’ digital risk perceptions, and individual digital resilience, thereby further strengthening the empirical research system in this field. Annual indicators covering 2007–2017 are subject to high sampling volatility due to small yearly coded policy records; aggregated stage-level comparison is preferred over fine-grained interpretation for individual early-year observations.

## Figures and Tables

**Table 1:** Policy text screening results and analytical sample.

Category	Raw Texts	Core Pool	Backup Review	Excluded Records	Annual Dimension Records
B1 Digital capability and age-friendly support	562	32	0	530	32
B2 Digital health management support	660	65	0	595	65
B3 Home- and Community-Based Care Support	793	272	139	382	271
B4 Institutional care and risk governance	1417	639	351	427	630
Total	3432	1008	490	1934	998

Note: The 998 annual dimension records differ from the 1008 core-pool records because 10 records lacked reliable year information or fell outside the annual counting window.

**Table 2:** Definitions and analytical relationship of B1–B4 policy-support dimensions and M1–M4 intervention mechanisms.

Code	Category	Main Identified Content	Corresponding Older-Adult Digital Behavioral Problem	COM-B and Risk-Related Dimension
B1	Digital capability and age-friendly support	Digital literacy, smart training, etc.	Poor operation, weak understanding, etc.	Capability and Risk Recognition
B2	Digital health management support	Telemedicine, chronic monitoring, etc.	Difficult data reading, medical procedure barriers, etc.	Capability and Health Self-management
B3	Home- and Community-Based Care Support	Day care, community facilities, etc.	Shortage of nearby help, limited access, etc.	Opportunity
B4	Institutional care and risk governance	Institutional supervision, safety standards, etc.	Mandatory online process, privacy threats, etc.	Risk Governance and Opportunity
M1	Capability enhancement (C)	Operational coaching, anti-fraud courses, etc.	Insufficient skills, poor risk judgment, etc.	Capability Enhancement
M2	Opportunity facilitation (O)	Service sites, family aid, etc.	Lack of service channels, no backup support, etc.	Opportunity Facilitation
M3	Motivation reinforcement (M)	Trust guidance, activity incentives, etc.	Tech anxiety, low willingness to use, etc.	Motivation Reinforcement
M4	Regulative risk governance	Data protection, emergency rules, etc.	System risks, personal online hazards, etc.	Regulative Risk Governance

**Table 3:** Representative evaluation indicators for LDA topic-number selection.

Number of Topics (K)	α	β	Perplexity	UMass Coherence	NPMI-Based Coherence Proxy	Manual Interpretability
5	0.2000	0.2000	525.948	−0.411	0.391	Overly broad; several governance themes merged
6	0.1667	0.1667	508.304	−0.326	0.353	Partly separated but insufficient differentiation
7	0.1429	0.1429	496.179	−0.436	0.358	Final selection: best balance between fit and interpretability
8	0.1250	0.1250	478.820	−0.434	0.345	Lower perplexity but topic fragmentation begins

Note: The full expanded comparison from K = 5 to K = 20 is reported in Table A1 in Appendix A. K = 7 was retained not because it produced the lowest perplexity, but because it provided the best balance between statistical fit, coherence, and substantive policy interpretability. The NPMI-based coherence proxy is used as an auxiliary interpretability-oriented indicator rather than as standard C_v coherence.

**Table 4:** B1–B4 structure in the core sample.

Code	Policy Support Dimension	Records	Proportion	Behavior-Oriented Meaning
B1	Digital capability and age-friendly support	32	3.21%	Directly responds to deficiencies in operation, understanding, risk recognition, and digital companionship
B2	Digital health management support	65	6.51%	Responds to difficulties in online health management, telemedicine, and device data interpretation
B3	Home- and Community-Based Care Support	271	27.15%	Provides nearby service and community assistance scenarios
B4	Institutional care and risk governance	630	63.13%	Ensures institutional operation, safety, quality, and informatization supervision
Total	-	998	100.00%	-

**Table 5:** Seven LDA topics and their behavior-oriented interpretations.

Topic	Semantic Name	Representative Terms	Interpretation for Older Adults’ Behavior
T1	Safety and emergency risk governance	fire protection, safety, hidden danger, emergency, food	Macro-level risk governance is strong, but fraud, privacy, operational errors, and manual fallback need further inclusion
T2	Institutional access, operation, and fees	licensing, fees, admission, private, public	Establishes institutional order, but pays limited attention to mandatory online registration and older adults’ understanding difficulties
T3	Planning, financing, and smart service supply	planning, smart, intelligent, public construction, scheme	Platform and facility supply increases, but usability depends on training and assistance
T4	Community facilities and day care	community, street, center, care	Provides nearby digital assistance scenarios for rural, solitary, and low-education older adults
T5	Home-community reform and pilots	home-based, community, pilot, reform, responsible unit	Promotes service scenario innovation, but pilot projects need to be transformed into normalized support
T6	Quality monitoring, star-rating, and credit	rating, star rating, spot check, on-site, credit	Should incorporate age-friendly experience, privacy protection, and manual fallback indicators
T7	Integrated medical-care and health services	medical-care integration, health care, health, nursing	Approaches health management needs, but must reduce barriers to understanding professional data

**Table 6:** Structure of dominant policy intervention mechanisms.

Mechanism	Main Content	Number of Documents	Proportion	Corresponding Behavioral Problem
M1 Capability enhancement	Training, education, operational guidance, health knowledge, anti-fraud and risk recognition	77	7.80%	Directly responds to not knowing how to use, not understanding, and not knowing how to identify risks
M2 Opportunity facilitation	Platforms, facilities, devices, service points, manual channels, volunteer and family support	122	12.36%	Provides access and assistance for rural, solitary, and disabled older adults
M3 Motivation reinforcement	Publicity, demonstrations, feedback, incentives, participation mobilization, trust building	212	21.48%	Reduces technology anxiety, failure expectations, and fear of fraud
M4 Regulative risk governance	Access, standards, inspections, credit, emergency response, data security, privacy protection	576	58.36%	Reduces institutional systemic risks and individual digital-use risks
Total	-	987	100.00%	-

**Table 7:** Comparison of B1–B4 structures in the core and extended samples.

Sample Scope	Sample Size Description	B1	B2	B3	B4	B3 + B4
Core sample	1008 core-pool records; 998 annual records	3.21%	6.51%	27.15%	63.13%	90.28%
Extended sample	Core pool + 490 backup review texts	2.14%	4.34%	27.44%	66.09%	93.53%

**Table 8:** Illustrative deductive framework for group-specific behavioral shortcomings, risk prevention, and digital resilience cultivation pathways; not derived from subgroup-specific statistical coding of policy texts.

Group	Core Behavioral Shortcoming	Risk-Prevention Objective	Digital Resilience Cultivation Pathway	Intervention Measure
Older adults with lower education or advanced age	Difficulty understanding procedures, inability to interpret health data, low self-efficacy	Reduce risks of operational errors, information misinterpretation, and insufficient fraud recognition	Develop understandable and repeatedly practicable digital health participation capability	Provide task-based training on online appointment registration, electronic medical insurance certificates, prescription inquiries, chronic disease data interpretation, and anti-fraud recognition; use large-font visuals, voice or dialect explanations, and on-site demonstrations.
Rural older adults	Insufficient network/devices, weak grassroots assistance, long travel distance	Reduce risks of inaccessibility to remote services and lack of assistance	Develop low-threshold, nearby, help-seeking service participation capability	Rely on township health centers, village clinics, and eldercare service stations to provide low-bandwidth access, mobile guidance, shared devices, and telephone plus on-site dual channels.
Older adults living alone	No one to ask for help, payment risk, fear of fraud, privacy concerns	Reduce risks of fraud, mistaken authorization, and lack of assistance after service interruption	Develop trusted reminders, follow-up support, and sustained participation in digital services	Set up community digital assistants, hotline call-back mechanisms, trusted contact authorization, abnormal transaction alerts, and privacy-protection prompts.
Disabled or semi-disabled older adults	Physical/cognitive limitations, inability to operate independently, lack of manual substitutes	Reduce risks of mandatory online procedures, unclear proxy responsibility, and insufficient privacy notification	Develop basic service protection that is proxy-enabled, substitutable, and accountable	Clarify caregiver proxy authorization, accessible interaction, institutional manual services, standards for online procedure substitutes, and privacy notification responsibilities.

Note: This stratified framework is deductively inferred from the COM-B model, overall coding results and existing aging digital divide literature. No subgroup statistical coding of policy texts was conducted to generate this table.

## Data Availability

The full original policy texts retrieved from PKULAW cannot be publicly redistributed because of database copyright and access restrictions. To support research transparency, the coding dictionary, keyword-search protocol, de-identified coded dataset, and analytical scripts for calculating DAPOI, BIDI, and LDA outputs are retained by the authors and are available from the first author upon reasonable formal request. The shared materials will be limited to metadata, coding labels, derived analytical variables, and calculation procedures necessary for academic verification of the main results.

## Abbreviations

The following abbreviations are used in this manuscript:

B1	Digital Capability and Age-Friendly Support
B2	Digital Health Management Support
B3	Home- and Community-Based Care Support
B4	Institutional Care and Risk Governance
BIDI	Behavioral Intervention Diversity Index
COM-B	Capability–Opportunity–Motivation–Behavior
DAPOI	Digital Adaptation Policy Orientation Index
LDA	Latent Dirichlet Allocation
M1	Capability Enhancement
M2	Opportunity Facilitation
M3	Motivation Reinforcement
M4	Regulative Risk Governance
PKULAW	Peking University Legal Database

## Appendix A

This appendix reports the expanded topic-number comparison used to support the final selection of K = 7 in the LDA model. Candidate topic numbers from K = 5 to K = 20 were compared using perplexity, UMass coherence, an NPMI-based coherence proxy, and manual interpretability. The results show that although perplexity generally decreases as K increases, higher K values produce increasingly fragmented and overlapping topics. Therefore, K = 7 was retained as the final model because it provided the best balance between statistical performance and substantive policy interpretability.

**Table A1:** Expanded comparison of LDA topic-number selection from K = 5 to K = 20.

K	α	β	Perplexity	UMass	NPMI-Based Coherence Proxy	Manual Interpretability
5	0.2000	0.2000	525.948	−0.411	0.391	Overly broad; several governance themes merged
6	0.1667	0.1667	508.304	−0.326	0.353	Partly separated but insufficient differentiation
7	0.1429	0.1429	496.179	−0.436	0.358	Final selection: best balance between fit and interpretability
8	0.1250	0.1250	478.820	−0.434	0.345	Lower perplexity but topic fragmentation begins
9	0.1111	0.1111	477.412	−0.504	0.372	Fragmentation increases
10	0.1000	0.1000	468.360	−0.500	0.395	Fragmentation increases
11	0.0909	0.0909	463.856	−0.560	0.350	Fragmentation increases
12	0.0833	0.0833	465.375	−0.493	0.376	Fragmentation increases
13	0.0769	0.0769	460.350	−0.490	0.386	Over-fragmented
14	0.0714	0.0714	448.363	−0.451	0.404	Over-fragmented
15	0.0667	0.0667	441.198	−0.450	0.376	Over-fragmented
16	0.0625	0.0625	443.502	−0.520	0.384	Over-fragmented
17	0.0588	0.0588	441.448	−0.518	0.428	Highly fragmented
18	0.0556	0.0556	445.558	−0.511	0.384	Highly fragmented
19	0.0526	0.0526	439.902	−0.589	0.369	Highly fragmented
20	0.0500	0.0500	433.854	−0.537	0.384	Highly fragmented
